# Development and psychometric testing of the family caregiver self-efficacy scale for patients in the early post-coronary artery bypass grafting

**DOI:** 10.1371/journal.pone.0314326

**Published:** 2025-02-12

**Authors:** Shiqi Zhou, Yinghong Zhang, Yuting Liu, Qi Yang, Pan Peng, Li Geng, Liu Hu

**Affiliations:** 1 Hubei Province Key Laboratory of Occupational Hazard Identification and Control, Institute of Nursing Research, School of Medicine, Wuhan University of Science and Technology, Wuhan, China; 2 Department of Cardiology, Wuhan Asian Heart Hospital, Wuhan, China; Shiraz University of Medical Sciences School of Medicine, ISLAMIC REPUBLIC OF IRAN

## Abstract

**Background and objectives:**

Family caregiver self-efficacy plays an important role in improving the health and quality of life of patients in the early post-coronary artery bypass grafting (CABG). However, there is a lack of targeted self-efficacy assessment tool for caregivers of patients. Thus, the purpose of this study was to develop a Family Caregiver Self-Efficacy Scale for patients in the early post-CABG (FCSES-EPCABG) and to test its reliability and validity.

**Methods:**

Based on self-efficacy theory, the initial scale was formed by the literature review, semi-structured interviews, Delphi expert consultation, and pre-survey. Through the convenience sampling method, 133 caregivers who met the selection criteria were chosen for the questionnaire survey at Wuhan Asian Heart Hospital from January 2024 to May 2024. The aim was to test the reliability and validity of the scale.

**Results:**

The final scale contained five dimensions of wound care, medication management, cardiac rehabilitation management, social support, and self-care, with a total of 22 items. The item-level content validity index ranged from 0.889 to 1.000, the scale-level content validity index/average was 0.985, and the content validity ratio ranged from 0.778 to 1.000. The exploratory factor analysis showed that the cumulative variance contribution rate of the five dimensions was 69.433%. In the criterion-related validity analysis, the total score of the FCSES-EPCABG was positively correlated with the total score of the General Self-Efficacy Scale (r = 0.762, *P*<0.001). The Cronbach’s alpha coefficient of the scale was 0.919, the half reliability was 0.779, and the test-retest reliability was 0.936.

**Conclusion:**

The FCSES-EPCABG has satisfactory reliability and validity, which is suitable for evaluating the self-efficacy of family caregivers of patients in the early post-CABG.

## Introduction

Coronary heart disease (CHD) is the leading cause of death worldwide [1,2]. Coronary artery bypass grafting (CABG) is a surgical procedure commonly used to treat CHD. It can help patients recreate vascular pathways, improve myocardial ischemia and hypoxia, and alleviate angina pectoris [3]. In China, about 50,000 people undergo CABG every year, and the number is growing [4]. In the early post-CABG (within 30 days after surgery) [5], patients suffer from many physical and psychological problems such as dyspnea, arrhythmia, fatigue, sleep disturbance, wound pain, leg swelling, depression, and anxiety [6]. These issues greatly increase the difficulty of caregiving.

Family caregivers play a vital role in the patients’ postoperative recovery. Not only are they involved in the patients’ daily life and therapeutic decision-making, but they are also the main source of financial and emotional support for the patients [7]. However, studies have shown that family caregivers are maladapted and overwhelmed when caring for patients with CABG. Caregivers have challenges such as managing surgical wounds, medication prevention, cardiac rehabilitation, and dietary guidance [8]. Thus, in addition to the need for relevant knowledge and skills to fulfill their caring role, family caregivers need to maintain a positive belief in their ability to care for patients in the face of these difficulties [9].

Caregiver self-efficacy refers to the beliefs caregivers hold about their capability of successfully performing particular caregiving tasks [10]. A high level of caregiver self-efficacy is essential for improving negative caregiving emotions, reducing psychological distress, decreasing caregiving burden, and enhancing caregiving competence [11,12]. In Bandura’s view, self-efficacy determined how much effort a person would put into a task and how long he could persevere when faced with difficulties [13]. There is evidence that confidence in performing certain tasks (self-efficacy) predicts performance on those tasks [14]. Some studies have shown that improving the caregivers’ self-efficacy is crucial to improving patients’ health and quality of life after cardiac surgery [15,16]. Assessing self-efficacy requires people to focus on specific tasks, which means that generic assessment tools may not provide specific information for clinical healthcare professionals [17]. Furthermore, Bandura also suggested that self-efficacy assessment tools should be designed for specialized domains [13].

Currently, self-efficacy assessment tools include general self-efficacy assessment scales and specific self-efficacy assessment scales. The General Self-Efficacy Scale (GSES) is generalizable and unable to assess the care issues for patients in the early post-CABG. Nowadays, there are specific self-efficacy assessment tools for caregivers of patients with dementia, cancer, and diabetes [14,18,19], but there is no self-efficacy assessment tool for caregivers of patients in the early post-CABG. The purpose of this study was to develop a Family Caregiver Self-Efficacy Scale for patients in the early post-CABG (FCSES-EPCABG) and to test its reliability and validity, thereby providing a measurement tool for assessing the level of self-efficacy of family caregivers and validating the effectiveness of self-efficacy interventions.

## Methods

**Fig 1:**
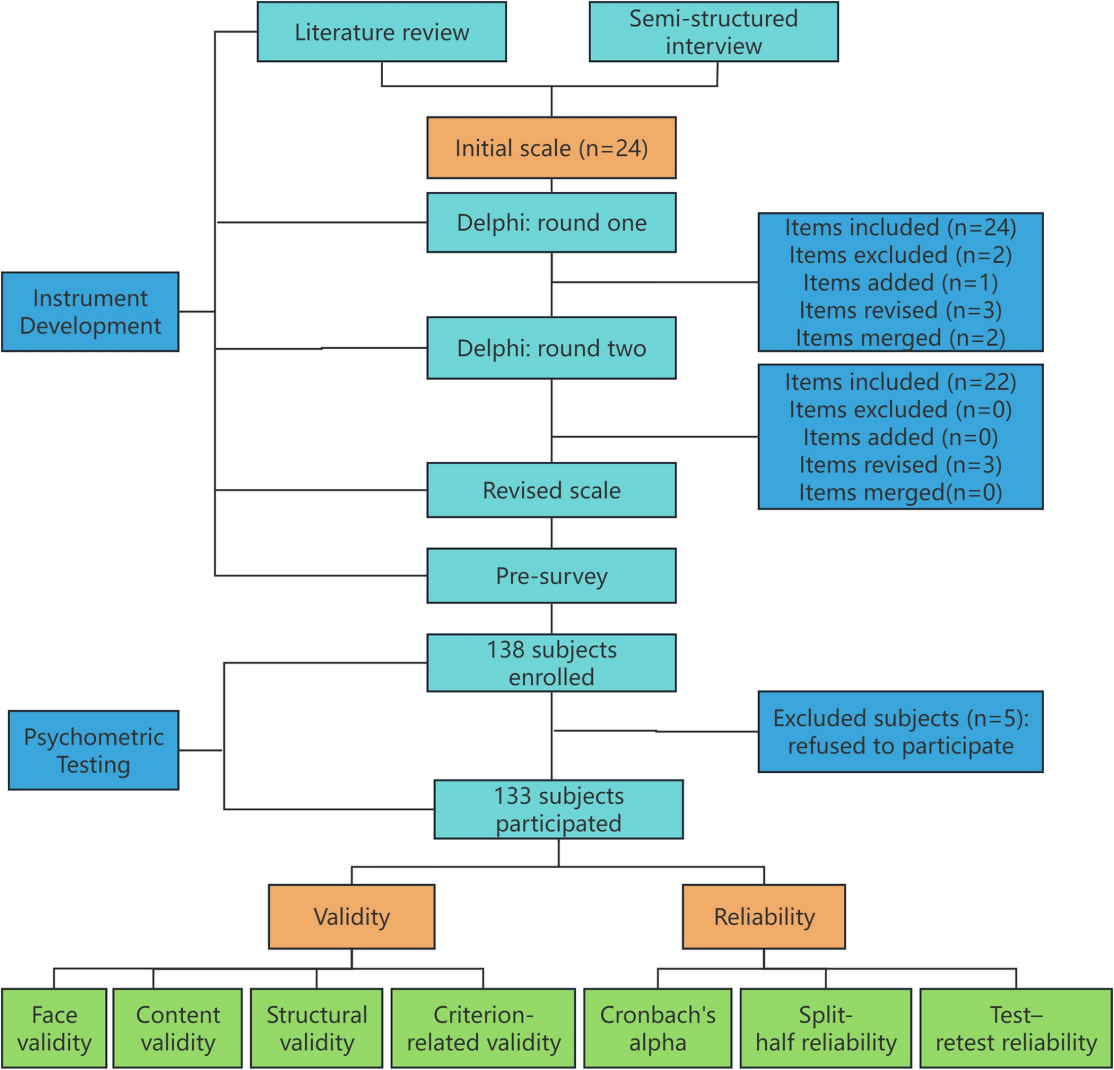
The process of developing the FCSES = EPCABG.

The study consisted of two phases, as illustrated in Fig 1: (1) developed the initial FCSES-EPCABG based on literature review, semi-structured interviews, Delphi expert consultation, and pre-survey. (2) tested the reliability and validity of the scale.

### Setting up a study team

The team consisted of seven members, including a cardiac surgeon, a nurse educator, a nurse manager, a clinical nurse, and three postgraduate students specializing in cardiovascular disease nursing. The main tasks of the team members included reviewing literature, conducting semi-structured interviews, organizing Delphi expert consultation, distributing and retrieving questionnaires, collecting data, and analyzing data.

### Literature review

The databases of PubMed, Embase, the Cochrane Library, Web of Science, CNKI, CBM, and Wanfang Data were searched. Search keywords were “Caregiver*, Family Caregiver*, Carer*”; “Self-Efficacy”; “Cardiovascular Disease*, Coronary Disease*, Coronary Heart Disease*, Coronary Artery Bypass*, Coronary Artery Bypass Grafting”. The retrieval time was from database inception to October 2023. The literature was screened and analyzed by reading the title, abstract, and full text to gather relevant content for this study. And then 15 items were preliminarily identified.

### Semi-structured interview

Using a phenomenological approach in qualitative research, the interview focused on the family caregivers’ experience and needs during the caring process. An initial interview outline was developed by reviewing the literature and consulting experts. Before the formal interviews, two family caregivers were selected for pre-interviews. Based on the results of the pre-interviews, the final interview outline was revised and refined. The interview outline was as follows: 1. How much do you know about caring for patients after CABG? 2. What difficulties have you encountered during the process of caring? How do you deal with them? 3. What help and support would you like? 4. How does caring for patients affect your work and life?

According to the inclusion and exclusion criteria of the formal survey, 12 caregivers of CABG patients were selected for interviews through convenience sampling in Wuhan Asian Heart Hospital in November 2023. Before the interview, informed consent and permissions were obtained from all participants whose interview materials would be collected. They signed a written informed consent. The location of the interviews was chosen to be in a quiet and private office. During the interview, we listened carefully and adapted the interview skills to the specific situation. Interviews lasted 20–30 minutes each. Stop the interview if nothing new came up.

Transcription of audio recording materials into text materials was conducted within 24 hours after completing interviews. Data was analyzed using the Colaizzi seven-step analysis method [20]: ①Read the interview materials carefully and repeatedly; ②Analyse information literally and identify and extract relevant, important, and meaningful statements to the research topic. ③Encode recurring viewpoints; ④Collect the viewpoints and distill meaningful common concepts to form the prototype themes; ⑤Each prototype theme was described in detail; ⑥Repeatedly compare to identify similar ideas and then construct themes; ⑦Themes were returned to respondents for confirmation to ensure the accuracy of the results.

The age of participants at this stage was 34–71 (58.00±9.98) years, and 58.33% were male. Half of the participants were children of the patients. Finally, three themes were developed, namely, feeling powerless when caring for patients (lacking wound care skills, lacking medication knowledge, and being confused about cardiac rehabilitation), perceiving social support, and lacking self-care awareness. Analysis of the interview data led to the addition of nine items and the revision of five items. In conclusion, the FCSES-EPCABG has been developed with 24 items in five dimensions: wound care, medication management, cardiac rehabilitation management, social support, and self-care.

### Delphi expert consultation

#### Selection criteria for experts

Experts were selected according to the following criteria: (1) had at least 10 years of relevant work experience; (2) had a title of intermediate level or above; (3) had a bachelor’s degree or above; (4) worked in areas related to clinical medicine, clinical nursing, nursing management, and nursing education in cardiac surgery.

#### Developing consultation questionnaires and conducting expert consultation

The Delphi expert consultation questionnaire consisted of three parts: (1) The item importance rating scale. The importance of each item was scored by the 5-point Likert scale ranging from 1 (very unimportant) to 5 (very important). The item selection criteria were as follows: the mean importance score of the item was 3.5 and above, and at the same time, the variation coefficient of the item was 0.25 and below. (2) The general information of experts. (3) Expert’s familiarity with the content of the consultation and the criteria for making judgments. Consultation questionnaires were distributed by e-mail and experts were requested to respond within 2 weeks. In the end, the 22-item scale was produced by combining expert opinions on merging, deleting, and modifying items.

### Pre-survey

Twenty caregivers of post-CABG patients were purposely sampled from Wuhan Asian Heart Hospital for the pre-survey. Adjust the presentation and wording of some items based on caregivers’ feedback. At the same time, we tested the quantitative and qualitative face validity.

To assess qualitative face validity, twenty caregivers were asked to give their views on the items’ suitability, difficulty, and clarity. To evaluate quantitative face validity, they were requested to assess the significance of the items on a 5-point Likert scale from 1 point (not important) to 5 points (quite important). The frequency × importance formula was used to calculate the impact factor of the items. Items were considered appropriate if they had an impact factor greater than 1.5 [21].

### Psychometric testing

#### Participants

From January 2024 to May 2024, researchers conducted in-person recruitment activities to recruit family caregivers who accompanied patients during their postoperative hospital stay from the Department of Cardiac Surgery, Wuhan Asian Heart Hospital. The questionnaire was given to the caregiver during the period from one week after the patient’s surgery to just before discharge. A total of 138 questionnaires were distributed and 133 respondents agreed to participate and completed the questionnaires. The response rate was 96.38%. The language used in the questionnaire was the local language (Mandarin).

The inclusion criteria included the following: (1) patients were diagnosed with coronary artery disease and underwent CABG for the first time; (2) patients aged 18 years or older; (3)family caregivers who were the patients’ spouse, child, or other family members aged 18 years or older; (4) if there was more than one caregiver at the same time, the primary caregiver would be chosen; (5) caregivers had good comprehension and expression skills.

The exclusion criteria included the following: (1) caregivers had an employment relationship with the patients; (2) caregivers had serious physical or mental illness such as malignant tumor, acute renal failure, schizophrenia and so on.

#### Sample size

The sample size for factor analysis was at least 5 to 10 times the number of items [22], taking into account 20% of invalid questionnaires, and a minimum sample of 132 cases was required. The test-retest reliability was assessed by selecting 15 participants and retesting them 2 weeks later.

### Instruments

1. General information questionnaire: including gender, age, occupation, education level, etc.
2. Family Caregiver Self-Efficacy Scale for patients in the early post-CABG. It contained 22 items with five dimensions. Each item was rated on a 5-point Likert scale ranging from 1 (not at all confident) to 5 (complete confidence), with higher scores implying higher levels of self-efficacy.
3. General Self-Efficacy Scale. The GSES was used to test the criterion-related validity. In the 1980s, Professor Schwarzer and others developed the GSES [23], and the Chinese version was developed jointly by scholar Zhang and Professor Schwarzer in 1995. There were 10 items in the scale. Cronbach’s alpha coefficient was 0.87, split-half reliability was 0.90, and retest reliability was 0.83.

#### Item analysis

The critical ratio method, correlation coefficient method, and Cronbach’s alpha coefficient method were used for the item analysis. The criteria were as follows: (1) critical ratio method: the total scores were ranked in descending order, with the top 27% as the high subgroup and the bottom 27% as the low subgroup. Two samples were tested to assess item discrimination, and critical ratios were 3.00 and above, indicating that items were poorly discriminated and considered for deletion. (2) Correlation coefficient method: examined the correlation between the score of each item and the total score of the scale. If the item-total correlation coefficients were less than 0.40, the items were considered for deletion. (3) Cronbach’s alpha coefficient method: if Cronbach’s alpha coefficient of the total scale increased significantly after the item was deleted, then the item would be deleted [22].

#### Validity analysis

Content validity: Nine experts were invited to assess the content validity of the scale. The three key judgment indicators were Item-level Content Validity Index (I-CVI), Scale-level Content Validity Index/Average (S-CVI/Ave), and Content Validity Ratio(CVR). The four-point Likert scale was used for scoring, with scores from one point to four points representing “irrelevant” to “very relevant”. I-CVI was the ratio of the number of experts scoring 3 or 4 to the total number of experts. S-CVI/Ave was the mean of I-CVI for all items. The scale was considered good content validity when the I-CVI was 0.78 and above, and the S-CVI/Ave was 0.90 and above [24]. To evaluate CVR, experts were requested to rate each item on a three-point scale, namely “unnecessary”, “useful but not necessary”, and “necessary”. Items with a CVR of 0.75 and above were retained based on the Lawshe table [25].

Structural validity: Bartlett’s test of sphericity was performed first, and exploratory factor analysis (EFA) was considered appropriate if the Kaiser-Meyer-Olkin (KMO) coefficient was greater than 0.60 with a *P* of less than 0.05. Principal component analysis (PCA) and varimax rotation were used to extract common factors. Each factor contained at least 3 items and the items had loading values were 0.5 and above on the factor to which they belonged. All common factors explained above 40% of the overall variation, indicating good structural validity of the scale.

Criterion-related validity: The GSES was used as the criterion and the criterion-related validity was evaluated based on the correlation coefficient.

#### Reliability analysis

Reliability analysis was conducted by assessing Cronbach’s alpha coefficient, split-half reliability, and test-retest reliability. The criteria were as follows: (1) Cronbach’s alpha coefficients were 0.700 and above indicating the high internal consistency of the scale. (2) The scale was divided into 2 subscales according to the odd-even numbering of the scale items, and correlation coefficients were calculated separately for each dimension and between the 2 subscale scores. The split-half reliability coefficients were 0.700 and above, indicating good split-half reliability. (3) After 2 weeks, 15 previous caregivers were randomly selected for re-measurement to assess the test-retest reliability of the scale. Retest reliability coefficients were 0.700 and above, indicating good stability of the scale across time [26].

### Statistical methods

The SPSS 26.0 was used to analyze the data of this study. Continuous variables were described using means and standard deviations (SD). Categorical variables were described using frequencies and percentages. The reliability of the Delphi expert consultation results was measured by the positive coefficient, authority coefficient, and Kendall’s concordance coefficient of the experts. Differences were considered statistically significant at *P*<0.05.

### Ethical consideration

All participants were informed of the purpose and significance of the study and completed the questionnaire anonymously after signing the written informed consent. This study protocol was approved by the Ethics Committee of the School of Medicine, Wuhan University of Science and Technology (No. 2023149).

## Results

### Results of Delphi expert consultation

A total of 16 experts were included who were aged 31 to 52 (41.81±7.01) years. Two experts had a doctoral degree, six experts had a master’s degree, and eight experts had a bachelor’s degree. They worked for 10 to 28 (18.06±6.17) years. There were six doctors, seven nurses, two nursing managers, and one nursing educator.

The recovery rates of the two rounds of expert consultation were both 100%. This indicated high levels of experts motivation. In the first round, the authority coefficient and Kendall’s concordance coefficient were 0.863 and 0.428, respectively. In the second round, the authority coefficient and Kendall’s concordance coefficient were 0.885 and 0.132, respectively.

In the first round of expert consultation, six experts proposed amendments. Based on the criteria for deleting items and expert opinions, two items were deleted, two items were merged, one item was added and the language of several items was revised. The details were as follows: (1) Two items were deleted because their importance scores were less than 3.50. They were: item 14 “I can encourage the patient to maintain a normal weight” and item 15 “I can create a quiet and comfortable sleeping environment for the patient”. (2) According to the experts’ opinion, it was proposed to merge item 11 “I can recognize the patient’s bad moods in time” and item 12 “I can help the patient to regulate their bad moods” into one item “I can recognize and help the patient to adjust bad emotions in time”. (3) Added an item “I can remind the patient not to do anything that might pull on the wound”. (4) Some of the items were adapted and modified as follows: change item 8 “I know what the patient can and cannot eat” to “I can follow the advice of medical staff to manage the patient’s diet”. For item 16, change “I can urge the patient to stop smoking” to “I can urge the patient to change unhealthy lifestyle habits such as smoking, alcohol abuse, staying up late, etc”. Change item 20 “My family can help me to take care of the patient” to “I can get support and help from other family members”. The second round of expert consultation questionnaire was developed by revising and refining the items.

In round two, no items were deleted and only the wording of three items was modified. After 2 rounds of expert consultation, the FCSES-EPCABG was developed with five dimensions and 22 items, including wound care (5 items), medication management (3 items), cardiac rehabilitation management (7 items), social support (4 items), and self-care (3 items).

### Pre-survey results

In the pre-survey, we conducted face validity testing with 20 participants, and the wording of two items was considered incomprehensible. After discussion in the study group and participants’ suggestions, the wording of the two items was modified. The evaluation of quantitative face validity revealed that none of the items had an impact factor of less than 1.5. No items were deleted, and 22 items were retained. The participants at this stage were 22–65 (47.25±12.48) years, and 55% were female.

### Psychometric testing

#### The general demographic characteristics of caregivers

A total of 138 questionnaires were distributed and 133 respondents agreed to participate and completed the questionnaire. The response rate was 96.38%. Overall, the majority of participants were female (58.65%). Nearly half were aged 45–59 years(48.12%). The demographic characteristics of caregivers are shown in Table 1 (see S1 Data for original data).

**Table 1:** Demographic characteristics of the caregivers (n = 133).

**Characteristics**	**Category**	**n (%)**
Gender	Male	55 (41.35)
	Female	78 (58.65)
Age (years)	<45	24 (18.05)
	45–59	64 (48.12)
	≥60	45 (33.83)
Occupation	Worker	30 (22.56)
	Farmer	34 (25.56)
	Freelance work	38 (28.57)
	Unemployed person	15 (11.28)
	Retirement	16 (12.03)
**Characteristics**	**Category**	**n (%)**
Education level	Primary school or lower	25 (18.80)
	Junior high school	61 (45.86)
	Senior high school	31 (23.31)
	Junior college or above	16 (12.03)
Monthly per capita household income (RMB)	<1000	10 (7.52)
	1000–2999	57 (42.86)
	3000–4999	43 (32.33)
	≥5000	23 (17.29)
Chronic disease	Have	42 (31.58)
	Have not	91 (68.42)
Relationship with the patient	Spouse	80 (60.15)
	Son/Daughter	45 (33.83)
	Sibling	5 (3.76)
	Other Relative	3 (2.26)
Caregiving time per day(hours)	<4	6 (4.51)
	4–8	33 (24.81)
	8–12	46 (34.59)
	>12	48 (36.09)

#### Item analysis

The results of the critical ratio method showed that the critical ratios were 5.240~10.578, and the differences between the items in the high and low groups were statistically significant (*P* < 0.001), with good differentiation of the items. The correlation coefficients between the items and the total score ranged from 0.482 to 0.725, and the correlation coefficients were significant (*P* < 0.001). The Cronbach’s alpha coefficient of the scale was 0.919, and no items were deleted that significantly increased the Cronbach’s alpha coefficient of the scale. In conclusion, no items were deleted, leaving 22 items.

#### Validity analysis

Content validity: Nine experts were randomly selected from the experts consulted in the Delphi expert consultation to evaluate the content validity of the scale. According to the score, the CVI was 0.889~1.000, the S-CVI/Ave was 0.985, and the CVR was 0.778~1.000, indicating acceptable content validity.

Structural validity: The KMO value of the scale was 0.864, and Bartlett’s sphere test reached a significant level (χ^2^ = 1766.454, *P* < 0.001). Using the maximum variance orthogonal rotation, the principal component analysis method set the standard as characteristic root >1, and five factors were obtained with a cumulative variance of 69.433%. The five factors were as follows: wound care (five items with the factor loading range of 0.656–0.796), medication management (three items with the factor loading range of 0.785–0.854), cardiac rehabilitation management (seven items with the factor loading range of 0.668–0.760), social support (four items with the factor loading range of 0.641–0.852), self-care (three items with the factor loading range of 0.823–0.844). Table 2 presents the results of the factorial load matrix of exploratory factor analysis.

**Table 2:** Factorial load matrix of exploratory factor analysis (n = 133).

Item	Wound care	Medication management	Cardiac rehabilitation management	Social support	Self- care
1. I can help the patient to care for chest and leg wounds properly, keeping them dry and clean.	**0.656**	0.332	0.238	0.119	0.106
2. I know the symptoms of the patient with an infected wound.	**0.747**	0.193	0.142	0.27	0.038
3. I can remind the patient not to do anything that might pull on the wound.	**0.745**	0.188	0.195	0.15	0.093
4. I can help the patient to tie the chest strap correctly.	**0.742**	0.158	0.190	0.028	0.169
5. I know how to relieve the patient’s leg oedema.	**0.796**	0.139	0.236	0.147	0.176
6. I know the effects of the patient’s medication.	0.322	**0.854**	0.208	0.096	0.126
7. I know the side effects and precautions of the patient’s medication.	0.308	**0.799**	0.233	0.135	0.179
8. I can help the patient develop the habit of taking medicine regularly and on time.	0.248	**0.785**	0.165	0.089	0.108
9. I can follow the advice of the medical staff to manage the patient’s diet after surgery.	0.200	0.066	**0.760**	0.061	0.066
10. I can help the patient to choose the appropriate form of active and passive rehabilitation exercise, avoiding sedentary and strenuous exercise.	0.296	0.166	**0.700**	0.026	0.158
11. I can urge the patient to complete the cardiac rehabilitation exercise program.	0.301	0.162	**0.737**	0.075	0.066
12. I can recognize and help the patient to adjust bad emotions in time.	0.033	-0.062	**0.683**	0.106	0.212
13. I can urge the patient to change unhealthy lifestyle habits such as smoking, alcohol abuse, staying up late, etc.	-0.019	0.360	**0.668**	0.116	0.006
14. I can help the patient to control blood pressure, blood glucose and lipids.	0.184	0.244	**0.692**	0.064	0.106
15. I can accompany the patient for recheck after the surgery.	0.190	0.072	**0.710**	0.149	0.102
**Item**	**Wound care**	**Medication management**	**Cardiac rehabilitation management**	**Social** **support**	**Self-** **care**
16. I can gain knowledge and skills about post-coronary artery bypass grafting care from hospital leaflets, books, the Internet, etc.	0.173	0.114	0.117	**0.783**	0.117
17. I can get support and help from other family members.	0.068	0.063	0.038	**0.641**	0.418
18. I will take the initiative to consult the medical staff when I encounter problems while caring for the patient.	0.135	0.159	0.148	**0.814**	0.028
19. I can seek social security by existing social welfare and insurance systems.	0.162	-0.017	0.117	**0.852**	0.154
20. I can coordinate my work, life, and caring tasks.	0.212	0.134	0.258	0.096	**0.823**
21. I can stay positive and optimistic.	0.160	0.097	0.239	0.200	**0.832**
22. I can maintain a regular diet and have enough sleep.	0.096	0.135	0.053	0.208	**0.844**

Note: Bold font indicates that the absolute value of loading is >0.5.

Criterion-related validity: The total score of the FCSES-EPCABG was positively correlated with the total score of the GSES (r = 0.762, *P*<0.001).

#### Reliability analysis

The Cronbach’s alpha coefficient of the total scale was 0.916, and the five dimensions’ Cronbach’s alpha coefficients ranged from 0.814 to 0.899. The split-half reliability coefficient of the total scale was 0.779, and the five dimensions’ split-half reliability coefficients ranged from 0.841 to 0.867. The test-retest reliability coefficient of the scale was 0.936, and the five dimensions’ test-retest reliability coefficients ranged from 0.737 to 0.899. The detailed information is shown in Table 3.

**Table 3:** Reliability of the scale.

Factor	Cronbach’s alpha coefficient	Split-half reliability	Test-retest reliability
Total scale	0.919	0.779	0.936
Wound care	0.868	0.867	0.899
Medication management	0.899	0.841	0.737
Cardiac rehabilitation management	0.872	0.848	0.878
Social support	0.814	0.861	0.843
Self-care	0.882	0.863	0.780

## Discussions

### The FCSES-EPCABG is scientific and reliable

Based on self-efficacy theory, this study focused on the group of family caregivers of patients in the early post-CABG and constructed a comprehensive pool of items based on literature review and semi-structured interviews. A total of 16 experts were invited to take part in two rounds of the Delphi expert consultation. The expert authority coefficients of the 2 rounds of Delphi expert consultation were 0.863 and 0.885 respectively. The high authority and motivation of the experts ensured the reliability of the Delphi expert consultation, and the legibility of the items was ensured by the pre-survey. In addition, questionnaire surveys were used to test the reliability and validity of the scale. Finally, a scale with five dimensions and 22 items was formed. During this process, we have considered the purposiveness, operability, and scientificity to ensure the preciseness and rationality of the scale.

### The FCSES-EPCABG has good reliability and validity

The reliability and validity of the scale were measured. The Cronbach’s alpha coefficient of the scale was 0.919, and the split-half reliability coefficient was 0.779. This showed that the internal consistency of the scale was high. The test-retest reliability of the scale was 0.936, which was greater than 0.700, indicating that the scale had good stability.

The S-CVI/Ave was 0.985, the I-CVI was 0.889~1.000, and the CVR was 0.889~1.000, indicating that the scale had good content validity. The exploratory factor analysis results showed that each item had a high load on one factor while a small load on other factors. All factors had a cumulative variance contribution rate of 69.433%. The loading of the items on the corresponding dimensions ranged from 0.641 to 0.854, indicating good structural validity. The GSES was used as the indicator of criterion-related validity, and the correlation coefficient between the total score of the FCSES-EPCABG and the total score of the GSES was 0.762 (*P* < 0.001), which indicated that the criterion-related validity of this scale was more desirable.

In conclusion, this scale has good reliability and validity to accurately and realistically assess the family caregiver self-efficacy of patients in the early post-CABG.

### The FCSES-EPCABG has strong clinical utility

Although the GSES is the most widely used self-efficacy scale in clinical research, it is a general scale and could not be targeted to assess the self-efficacy of family caregivers of patients in the early post-CABG. Researchers believed that measuring self-efficacy needed to target a specific field, and should represent the different task requirements in the field. The FCSES-EPCABG was designed in five dimensions: wound care, medication management, cardiac rehabilitation management, social support, and self-care. Thus, this five-dimension scale can quantify the level of caregiver self-efficacy for patients in the early post-CABG.

The first dimension, wound care, was used to measure the family caregivers’ confidence in caring for the patients’ chest and leg wounds. Wound healing after CABG is a long process and early postoperative wound care often requires the involvement of family caregivers. For example, caregivers can assist the patient in tying the chest strap and sterilizing the wound. Margo’s research has shown that caregivers are concerned about the potential for infection in patients’ wounds [27]. Caregivers with high levels of self-efficacy are more likely to learn correct wound care ways, recognize the symptoms of wound infection, and treat them on time.

The second dimension, medication management, was used to measure family caregivers’ confidence in helping patients develop good medication habits. Despite the significance of secondary prevention, poor medication adherence has been reported in patients after CABG, with approximately 50% of patients failing to take their prescribed medications [28]. The higher the caregivers’ self-efficacy in medication management, the better the caregivers will be able to remind the patients to take the medication timely and correctly. And finally, it can improve the patient’s adherence to the medication.

The third dimension, cardiac rehabilitation management, was used to measure family caregivers’ confidence in assisting patients with cardiac rehabilitation management. Cardiac rehabilitation for post-CABG patients is an important adjunct to the surgical management of CABG. Rehabilitation programs including exercise, nutrition, psychology, and risk factor management are designed to help patients develop a healthy lifestyle [29]. Caregivers’ involvement in cardiac rehabilitation has been shown to improve self-management and physical activity levels in CABG patients.

The fourth dimension was social support, which measured the caregivers’ confidence in receiving social support when caring for the patient. Social support refers to the help a person receives from society, family and friends, and it can assist caregivers in meeting their emotional, material and financial needs [30]. High levels of perceived social support from family caregivers can increase caring motivation, and reduce the negative emotions of caring burden.

The fifth dimension, self-care, was used to measure the family caregivers’ confidence in self-care when caring for the patients. Family caregivers’ physical and mental health is the foundation of caregiving. Kathryn et al. [31] determined that the higher sense of self-care caregivers have, the better they can regulate negative emotions to care for patients.

The FCSES-EPCABG, an important assessment tool, can be used to scientifically and objectively assess the self-efficacy of family caregivers of early postoperative CABG patients, which helps healthcare professionals understand the self-efficacy level of family caregivers and implement targeted clinical interventions for them. The FCSES-EPCABG consists of five dimensions with a total of 22 items, a moderate number of scale items, and a completion time of 5–10 minutes. The items are clear, easy to understand and complete. Thus, this scale is very practical and has a high degree of clinical utility.

### Limitations

This study presents the following limitations. First, the study used the convenience sampling method to select data from the first five months of the year, however, it is well-established that depression, anxiety, and performance can vary by season, which may also influence caregiver confidence. Although psychological changes caused by seasons and weather are complex, their impact on caregivers’ self-efficacy should be considered in further surveys. In addition, the study was conducted in one hospital, which was a tertiary-level hospital specialized in cardiovascular diseases and was approved as the Hubei Province Clinical Nursing Key Specialty. Hence, its level of medical treatment and nursing care for CABG patients may be higher than that in other hospitals. What’s more, the majority of respondents were from Hubei Province. Therefore, it is recommended that the next studies should expand the sample size and study area to further validate the scale. Finally, the data were collected as self-reports, so respondents may overestimate or underestimate their competence, which may reduce the accuracy of the data.

## Conclusion

Based on the standardized scale development process, the FCSES-EPCABG was constructed. The scale which includes five dimensions with a total of 22 items has good scientific and practical validity and can be used as a measurement tool for healthcare professionals to assess the level of CABG patients’ family caregiver self-efficacy.

## Supporting information

S1 Data (XLSX)
